# Improved Electrical Characteristics of AlGaN/GaN High-Electron-Mobility Transistor with Al_2_O_3_/ZrO_2_ Stacked Gate Dielectrics

**DOI:** 10.3390/ma15196895

**Published:** 2022-10-05

**Authors:** Cheng-Yu Huang, Soumen Mazumder, Pu-Chou Lin, Kuan-Wei Lee, Yeong-Her Wang

**Affiliations:** 1 Department of Electrical Engineering, Institute of Microelectronics, National Cheng-Kung University, Tainan 701, Taiwan; 2 Department of Photonics, National Cheng-Kung University, Tainan 701, Taiwan; 3 Department of Electronic Engineering, I-Shou University, Kaohsiung 840, Taiwan

**Keywords:** metal-oxide-semiconductor (MOS), high-electron-mobility transistor (HEMT), Al_2_O_3_, ZrO_2_

## Abstract

A metal-oxide-semiconductor high-electron-mobility transistor (MOS-HEMT) is proposed based on using a Al_2_O_3_/ZrO_2_ stacked layer on conventional AlGaN/GaN HEMT to suppress the gate leakage current, decrease flicker noise, increase high-frequency performance, improve power performance, and enhance the stability after thermal stress or time stress. The MOS-HEMT has a maximum drain current density of 847 mA/mm and peak transconductance of 181 mS/mm. The corresponding subthreshold swing and on/off ratio are 95 mV/dec and 3.3 × 10^7^. The gate leakage current can be reduced by three orders of magnitude due to the Al_2_O_3_/ZrO_2_ stacked layer, which also contributes to the lower flicker noise. The temperature-dependent degradation of drain current density is 26%, which is smaller than the 47% of reference HEMT. The variation of subthreshold characteristics caused by thermal or time stress is smaller than that of the reference case, showing the proposed Al_2_O_3_/ZrO_2_ stacked gate dielectrics are reliable for device applications.

## 1. Introduction

In recent decades, AlGaN/GaN high-electron-mobility transistors (HEMTs) have received much attention due to their high breakdown voltage, high saturation current, and high operating temperature. These advantages make AlGaN/GaN HEMTs a potential candidate for high-frequency and high-power device applications [1,2,3]. However, conventional GaN-based HEMTs with a Schottky gate suffer from high leakage current and the current collapse effect, which degrade the device performance and reliability. Inserting dielectric layers underneath the metal gate can effectively reduce the gate leakage current [4,5], suppress current collapse phenomenon [6,7], provide better linearity of RF output power [8,9], and lower flicker noise [10,11]. As the size of the device shrinks, the thickness of the gate dielectric shrinks, resulting in leakage current or related reliability issues.

A thin oxide layer leads to large current leakage; however, high-κ materials increase the physical thickness of gate dielectrics and can thus overcome the tunneling leakage current. The high-κ materials such as HfO_2_ [12], TiO_2_ [13], ZrO_2_ [14], HfAlO_x_ [15], etc. are expected to increase the capacitance of the transistor without reducing the oxide thickness. Recent research has proposed that different oxide compounds have excellent dielectric properties [16]. Among these materials, ZrO_2_ is a potential choice. ZrO_2_ has a wide bandgap (5.16–7.8 eV) and a high dielectric constant (20–25) [17]. However, the ZrO_2_/GaN interface has some issues, such as a poor conduction band offset with GaN of (~1.1 eV), and a very high interface state density. An ultra-thin Al_2_O_3_ film can be inserted to suppress the interface charge of GaN. Al_2_O_3_ has a large bandgap (7–8.8 eV) that can tolerate a high breakdown field [18]. Based on the above discussion, stacked or bilayer gate dielectrics are the effective solution for modifying the interface quality. Strong correlation was observed between different compound contents and microwave dielectric properties [19,20]. Many studies have been widely explored, and excellent results have been obtained [21,22,23,24,25] for GaN-based HEMT; however, the reliability of Al_2_O_3_/ZrO_2_ on AlGaN/GaN HEMTs has rarely been evaluated [23].

Many reports have been published in the GaN-based areas and they often studied the gate leakage, dc, and C-V characteristics at room temperature or high temperature. However, flicker noise, high-frequency performance, power performance, and stability after thermal stress or time stress were less conducted. On the other hand, although stacked oxide layers are available in some studies, there are only a few on the combination of Al_2_O_3_ and ZrO_2_. Authors just studied the trapped charge effects for MOS capacitor or HEMT with Al_2_O_3_ or ZrO_2_ (i.e., single layer, not stacked layer). In this work, a radio frequency (RF) co-sputter system was used to deposit 1 nm-thick Al_2_O_3_ and 12 nm-thick ZrO_2_ films as a stacked gate dielectrics on AlGaN/GaN HEMT to increase breakdown voltage and reduce leakage current. In addition to low-frequency noise and high-frequency performance, the reliabilities of thermal and time stress for proposed devices were investigated.

## 2. Experimental Study

The wafer structure was grown with metal-organic chemical vapor deposition (MOCVD) on a 6-inch p-type (≤0.03 Ω·cm) Si-(111) substrate. The epi-structure from the top to bottom consisted of a 2-nm-thick GaN cap layer, a 20-nm-thick Al_0.25_Ga_0.75_N barrier layer, a 300-nm-thick GaN channel layer, and a 3.9-μm-thick buffer/transition layer. The sheet resistivity, sheet carrier concentration, and Hall mobility were 448.6 Ω/cm, 2338 cm^2^/V·s, and 6.137 × 10^12^ cm^−3^, respectively. Samples were cleaned in an ultrasonic machine with acetone, methanol, and deionized water for 5 min. For device fabrication, the mesa isolation was conducted using an inductive coupled plasma reactive ion etching (ICP-RIE) system under a Cl_2_ and BCl_3_ mixed-gas environment. Ohmic contact of source/drain regions were deposited with Ti/Al/Ni/Au (25 nm/150 nm/30 nm/120 nm) by electron beam evaporation and followed by rapid thermal annealing (RTA) at 875 °C for 45 s in N_2_ ambient. After that, the gate region was defined by photolithography. For reference Schottky-gate HEMT, gate electrode was deposited directly. For MOS-HEMT, the gate dielectrics of 1-nm Al_2_O_3_ and 12-nm ZrO_2_ stacked layer was then deposited by RF co-sputter system. The co-sputtering system ULVAC ACS-4000-C3 includes electrodes of two DC power supplies and one RF power source. The argon gas flow rate was 30 sccm and the RF power was 100 W for Al_2_O_3_ of 2 min and ZrO_2_ of 11 min, respectively. Finally, a gate electrode of Ni/Au (80 nm/100 nm) was deposited using the electron beam evaporation. The gate length (L_G_), gate width (W_G_), and source-to-drain distance (L_SD_) were 1 µm, 100 µm, and 5 µm, respectively. The dc characteristics of the proposed devices were measured using an Agilent B1500A semiconductor analyzer. The low-frequency noise spectrum was measured using a ProPlus system. NoisePro Plus software was used to extract low-frequency noise model parameters. S-parameter measurements of the high-frequency devices were performed by a Keysight N5245A PNA-X microwave network analyzer.

## 3. Results and Discussion

**Figure 1:**
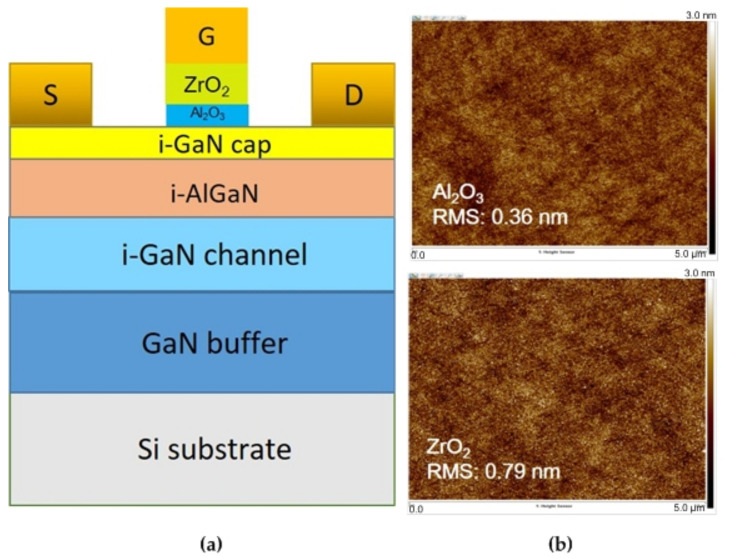

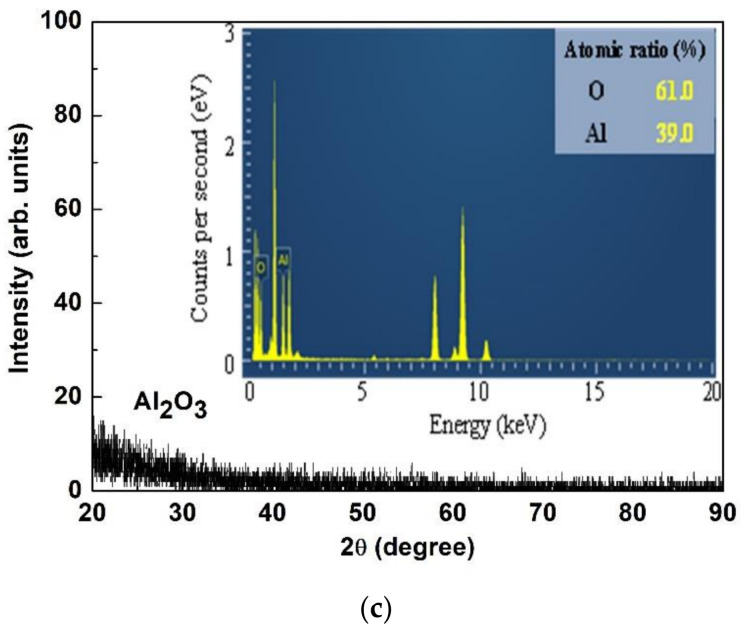

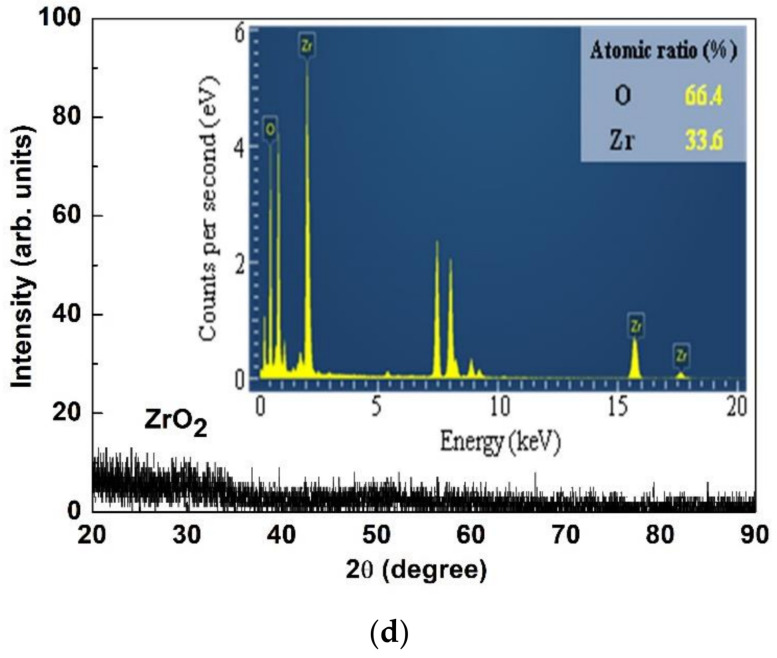

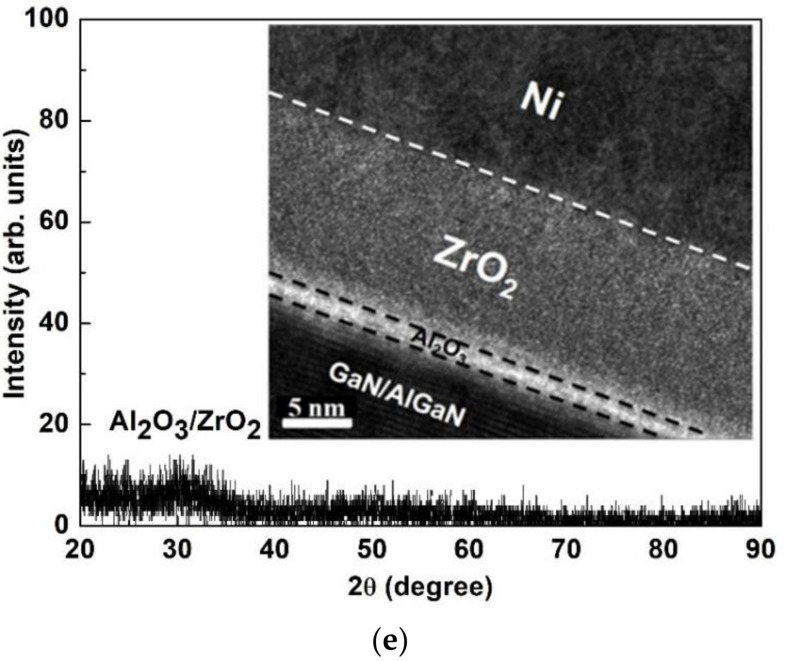
(**a**) Schematic structure of proposed MOS-HEMT; (**b**) AFM images of Al_2_O_3_ and ZrO_2_; (**c**–**e**) XRD spectra of the Al_2_O_3_, ZrO_2_, and Al_2_O_3_/ZrO_2_, respectively. The insets of (**c**,**d**) show the related EDS spectra. The inset of (**e**) shows the TEM image of the Al_2_O_3_/ZrO_2_ stacked layer.

A cross-sectional schematic diagram of the device is shown in Figure 1a. Figure 1b shows the atomic force microscopy (AFM) image (5 μm × 5 μm) of the Al_2_O_3_ and ZrO_2_. The root mean square (RMS) value of Al_2_O_3_ was 0.36 nm and that of ZrO_2_ was 0.79 nm, indicating that the surface roughness of oxide films is still good. Figure 1c–e show the X-ray diffraction (XRD) patterns of Al_2_O_3_, ZrO_2_, and Al_2_O_3_/ZrO_2_ stacked layer, respectively. The XRD patterns did not reveal any peaks corresponding to the single crystal or polycrystalline phases, indicating that the RF co-sputtered oxide films were amorphous. The diffraction peaks of AlGaN, GaN, and substrate are not measured due to the use of grazing incidence at a small angle. The insets of Figure 1c,d show the related energy-dispersive X-ray spectroscopy (EDS) spectra for Al_2_O_3_ and ZrO_2,_ respectively. The elemental composition ratio of the Al_2_O_3_ was nearly 2:3, and that of the ZrO_2_ was around 1:2. The inset of Figure 1e shows the transmission electron microscopy (TEM) image of the Al_2_O_3_/ZrO_2_ stacked layer between the metal and semiconductor. The image reveals that the Al_2_O_3_ and ZrO_2_ layers are not crystalline, which is consistent with the XRD pattern.

**Figure 2:**
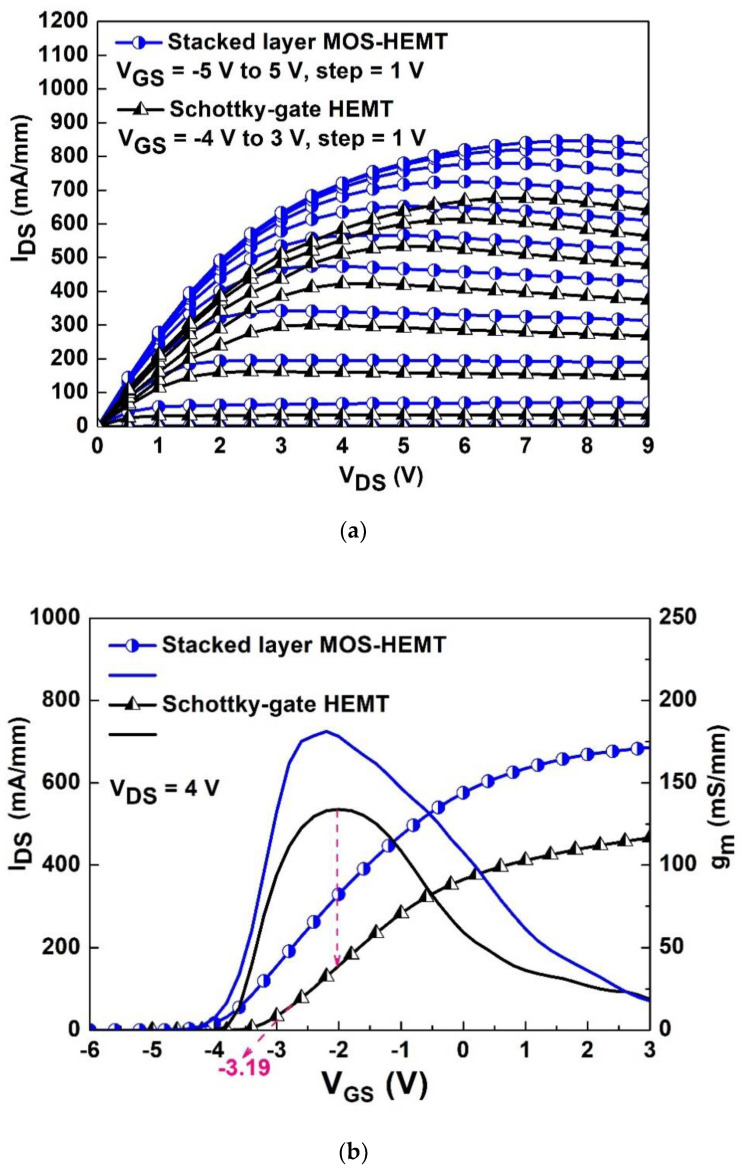
(**a**) I_DS_-V_DS_ characteristics and (**b**) corresponding g_m_ and I_DS_ versus V_GS_ at 4 V of V_DS_ for both devices.

Figure 2a shows the drain current density (I_DS_) versus drain voltage (V_DS_) characteristics of the Schottky-gate HEMT for reference and the proposed Al_2_O_3_/ZrO_2_ stacked-layer MOS-HEMT, respectively. The corresponding static on-state resistance (R_on_) are 6.21 Ω·mm and 3.82 Ω·mm at gate voltage (V_GS_) = 0 V in the linear region, respectively. The maximum I_DS_ of the stacked layer MOS-HEMT is 847 mA/mm at V_GS_ = 5 V, which is higher than that (676 mA/mm at V_GS_ = 3 V) of the Schottky-gate HEMT owing to the high-κ oxide layer. Figure 2b shows the comparison of transconductance (g_m_) and I_DS_ of Schottky-gate HEMT and stacked-layer MOS-HEMT at 4 V of V_DS_. The g_m_ values are 134 mS/mm and 181 mS/mm, respectively. The corresponding threshold voltages (gate swing voltages) are −3.19 V (2.21 V) and −3.88 V (3.02 V), respectively. The threshold voltage is determined as the V_GS_ intercept of the linear extrapolation of the drain current at the point of peak g_m_. For example, the red dotted line in Figure 2b intersects the horizontal axis. The deposited Al_2_O_3_/ZrO_2_ layer makes the threshold voltage of MOS-HEMT shift to the negative direction due to the charges at the interface.

**Figure 3:**
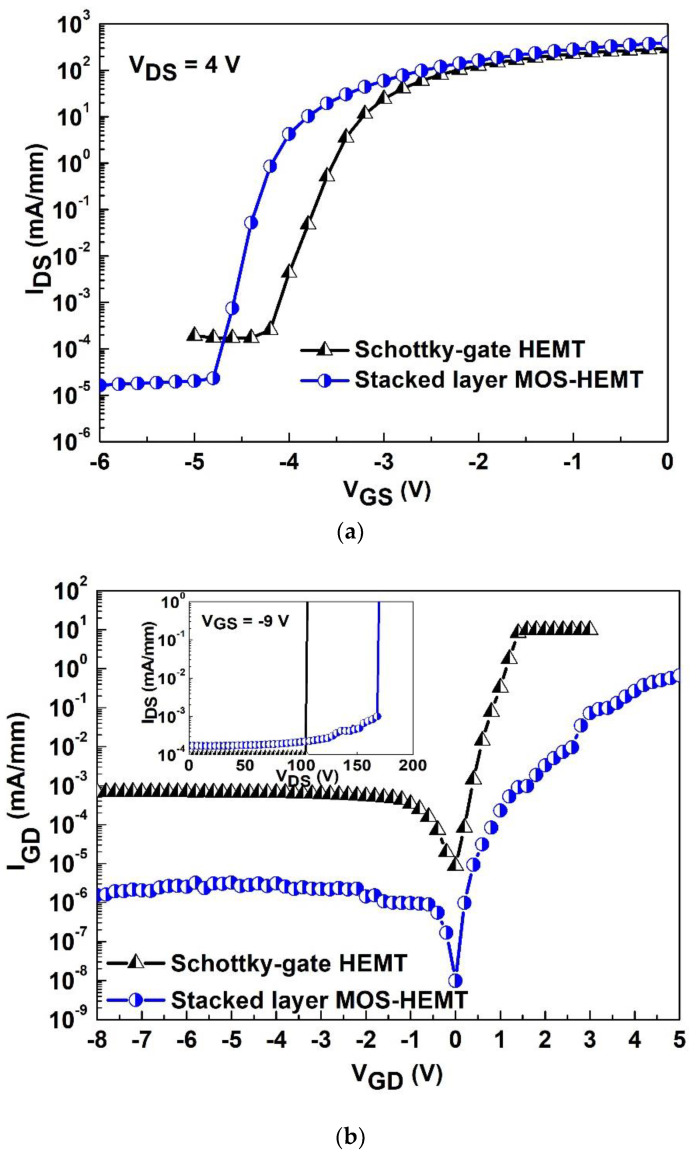
(**a**) Comparison of subthreshold current for the Schottky-gate HEMT and stacked layer MOS-HEMT. (**b**) Comparison of two-terminal gate leakage current for both devices. The inset shows three-terminal off-state breakdown voltage.

Figure 3a shows the comparison of subthreshold characteristics for the Schottky-gate HEMT and stacked-layer MOS-HEMT. The subthreshold swing (I_ON_/I_OFF_ ratio) values are 170 mV/dec (2.2 × 10^6^) and 95 mV/dec (3.3 × 10^7^) at V_DS_ = 4 V, respectively. The subthreshold swing is the reciprocal of the slope from the curve in Figure 3a, i.e., $\partial V_{\mathrm{GS}}/\partial \left({\mathrm{logI}}_{\mathrm{DS}}\right)$. The lower subthreshold swing can ensure excellent pinch-off and low power dissipation in digital applications, and good power-added efficiency in analog applications. Due to the Al_2_O_3_/ZrO_2_ between the gate electrode and GaN, the off-state I_DS_ is lower than that of the reference case. Figure 3b shows the two-terminal gate leakage current for the Schottky-gate HEMT and stacked-layer MOS-HEMT. The reverse gate leakage currents are 6.91 × 10^−4^ mA/mm and 1.75 × 10^−6^ mA/mm at V_GS_ = −8 V, respectively. The gate leakage current can be reduced by around three orders of magnitude for the MOS-HEMT due to the higher energy barrier between the gate metal and GaN. The related turn-on voltages are 1.6 V and more than 5 V, respectively. Similar results can be observed in the three-terminal off-state breakdown voltage for both devices as shown in the inset. The off-state breakdown voltages of the reference HEMT and Al_2_O_3_/ZrO_2_ MOS-HEMT are 104 and 170 V, respectively. The off-state breakdown voltage is defined as the drain voltage at the drain current density of 1 mA/mm. Therefore, the gate leakage current and breakdown can be suppressed through the MOS structure instead of the Schottky-gate structure.

**Figure 4:**
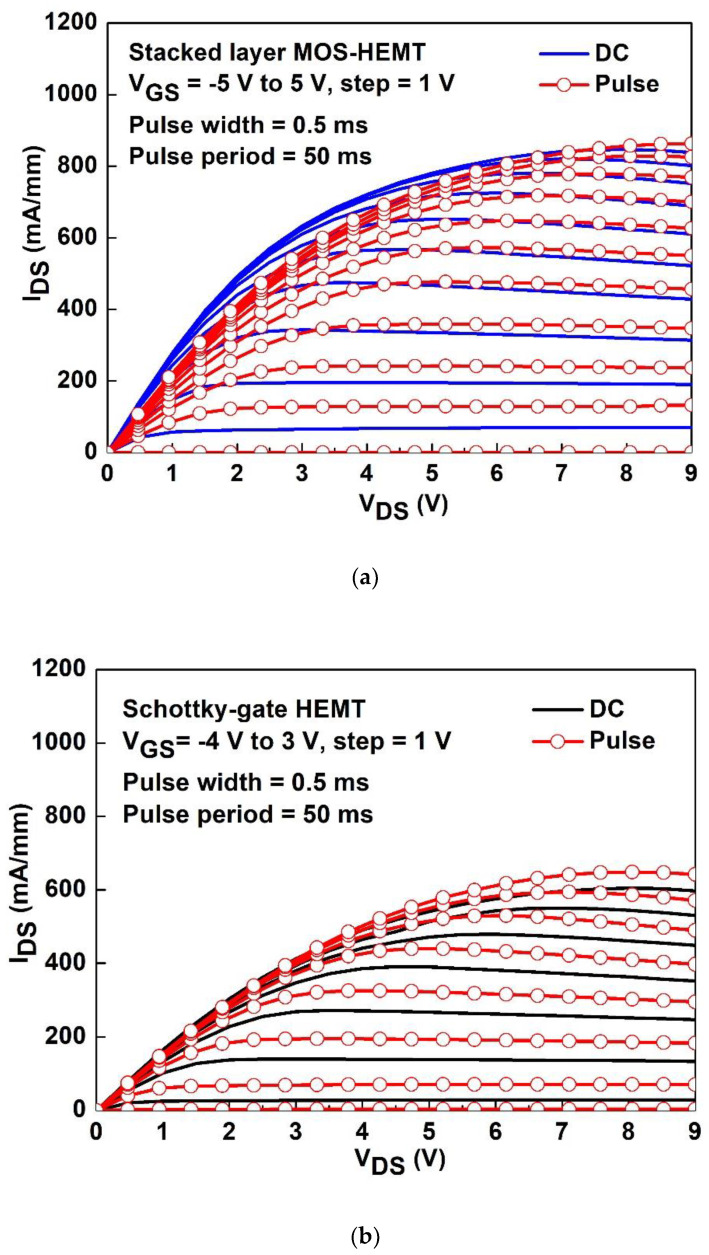

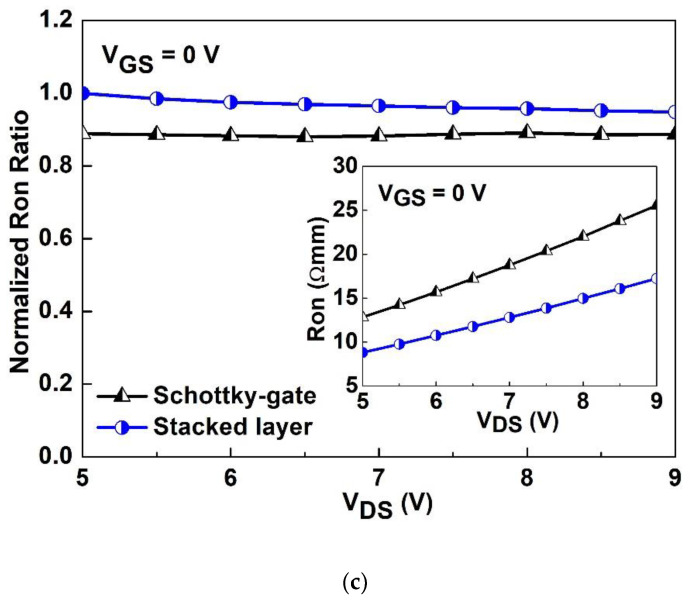
The pulse I_DS_-V_DS_ characteristics of the (**a**) Schottky-gate HEMT and (**b**) the Al_2_O_3_/ZrO_2_ MOS-HEMT. (**c**) The normalized dynamic R_on_ and related static R_on_ at V_GS_ = 0 V for both devices.

Figure 4a,b show the pulse I_DS_-V_DS_ characteristics of the Schottky-gate HEMT and stacked-layer MOS-HEMT, respectively. The pulse width is 0.5 ms and pulse period is 50 ms. Drain current degradation of the reference HEMT and the stacked-layer MOS-HEMT are approximately 8.1% and 2%, respectively. Figure 4c shows the normalized dynamic R_on_ ratio and the related static R_on_ at V_GS_ = 0 V for both devices. Under the condition of high V_DS_, the device with a stacked oxide layer has less drain current degradation than that of the Schottky-gate HEMT, so its normalized R_on_ is close to 1. Surface traps on the GaN are suppressed and passivated by the Al_2_O_3_/ZrO_2_, leading to the lower drain current degradation than with the reference HEMT. The degradation may be also caused by the interaction between the self-heating and trapping effect [26].

**Figure 5:**
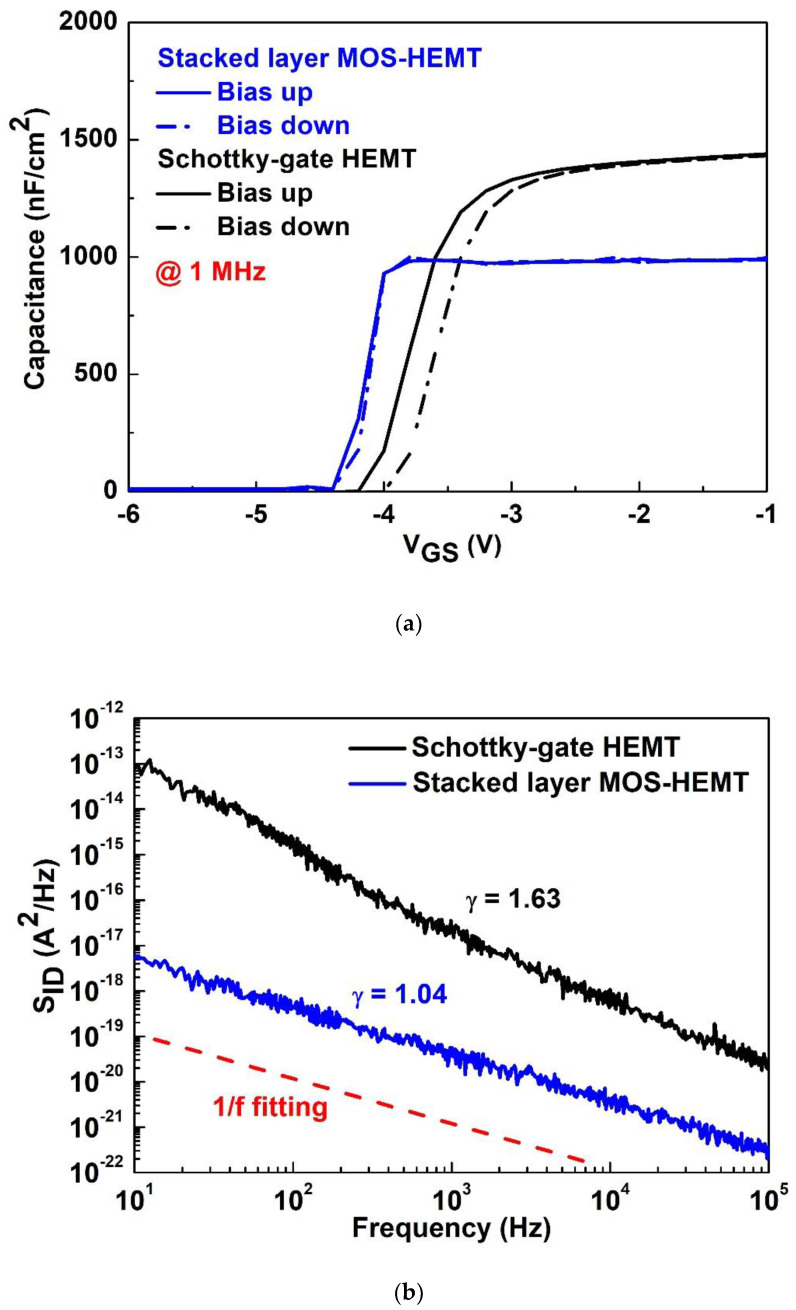
(**a**) 1 MHz C-V measurements and (**b**) flicker noise characteristics for both devices.

In order to have better insight into the impact of interface traps, high-frequency capacitance-voltage (C-V) and low-frequency noise (i.e., flicker noise) for both devices were measured, as shown in Figure 5a,b, respectively. When the up and down sweep lines do not coincide, the presence of hysteresis (ΔV) is mainly result from the interface traps. The interface trap density (D_it_) was calculated via the capacitance and ΔV from the high-frequency capacitance measurements [27]. The ΔV (D_it_) were 220 mV (1.92 × 10^12^ cm^−2^eV^−1^) for the Schottky-gate HEMT and 40 mV (2.44 × 10^11^ cm^−2^eV^−1^) for the stacked-layer MOS-HEMT. Lower flicker noise indicates fewer defects or traps at the interface between the metal gate and semiconductor. Defects or traps capture/emission charges via biases, and the total charges within the channel change, resulting in drain current degradation as shown in Figure 4. In addition, the frequency exponent (γ) was fitted as 1.63 and 1.04 for the reference HEMT and MOS-HEMT, respectively. A larger γ closely corresponded to generation-recombination center. Due to the stacked Al_2_O_3_/ZrO_2_ layer, the electric field strength around the gate-drain region is relatively small, resulting in less carrier scattering in the channel. Thus, the Al_2_O_3_/ZrO_2_ stacked layer reduces the gate leakage current at the interface and can also satisfy the dangling bonds on the GaN to suppress defects or traps, leading to reduced carrier scattering and flicker noise.

**Figure 6:**
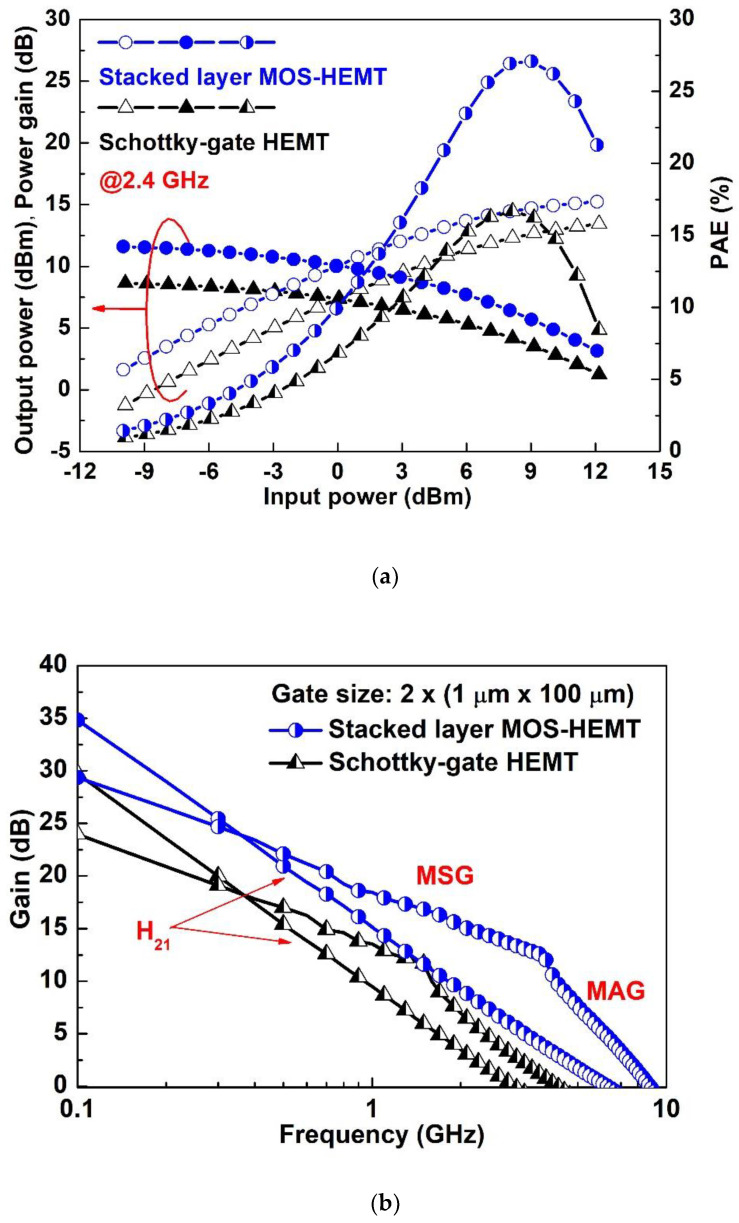
Comparison of (**a**) the microwave characteristics at maximum g_m_ and (**b**) the power performance as a function of the input power at 2.4 GHz for both devices.

Based on the S-parameter measurement in Figure 6a, the unity–current–gain cutoff frequency (*f_T_*) and the maximum oscillation frequency (*f_max_*) were 3 (6.4) GHz and 4.1 (9.1) GHz at maximum g_m_ for the Schottky-gate HEMT (MOS-HEMT). The increased microwave performances of the Al_2_O_3_/ZrO_2_ MOS-HEMT may be attributed to the increase in the ratio of g_m_ to gate-source capacitance or the addition of high-κ material [26]. Similar results were also observed in [28,29]. Figure 6b shows the comparison of output power, power gain, and power-added efficiency (PAE) versus input power at 2.4 GHz for both devices. The saturated output power, power gain, and maximum PAE are 13.4 (15.2) dBm, 8.6 (11.6) dB, and 16.7 (27.1)% for the reference HEMT (MOS-HEMT), respectively. The PAE can be supposed that the rate of input DC power is transformed into output AC power. Improved current drive, g_m_, and gate leakage current obtained in Al_2_O_3_/ZrO_2_ MOS-HEMT are beneficial to the PAE. Therefore, Al_2_O_3_/ZrO_2_ MOS-HEMT demonstrated better saturated output power and maximum PAE than those of the reference case.

**Figure 7:**
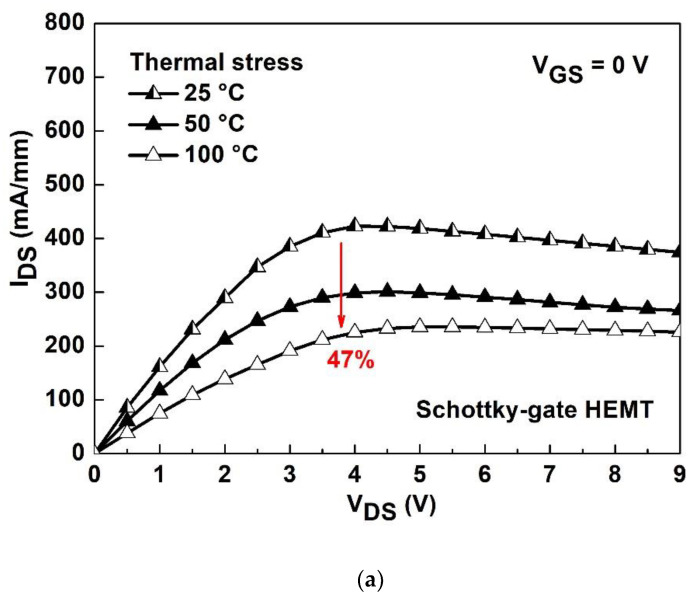

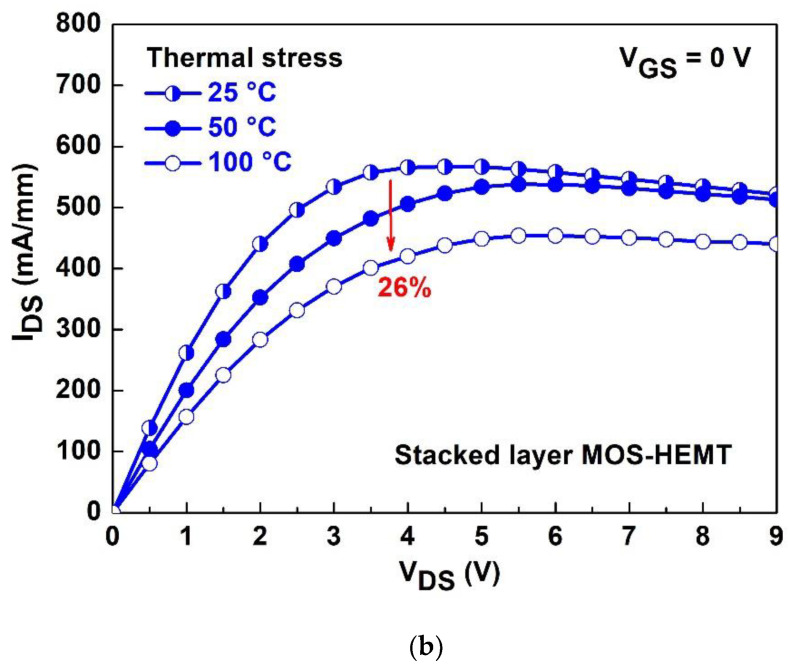
I_DS_-V_DS_ characteristics with various temperatures ranged from 25 °C to 100 °C for (**a**) the Schottky-gate HEMT and (**b**) the stacked layer MOS-HEMT.

**Figure 8:**
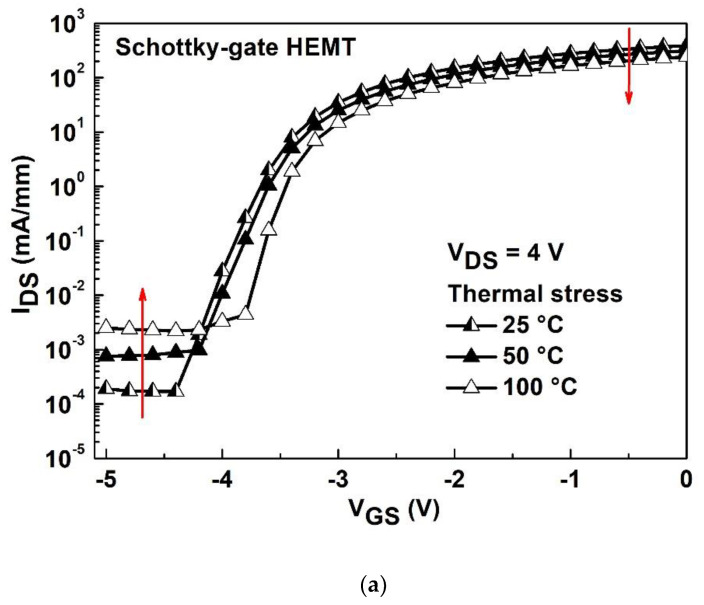

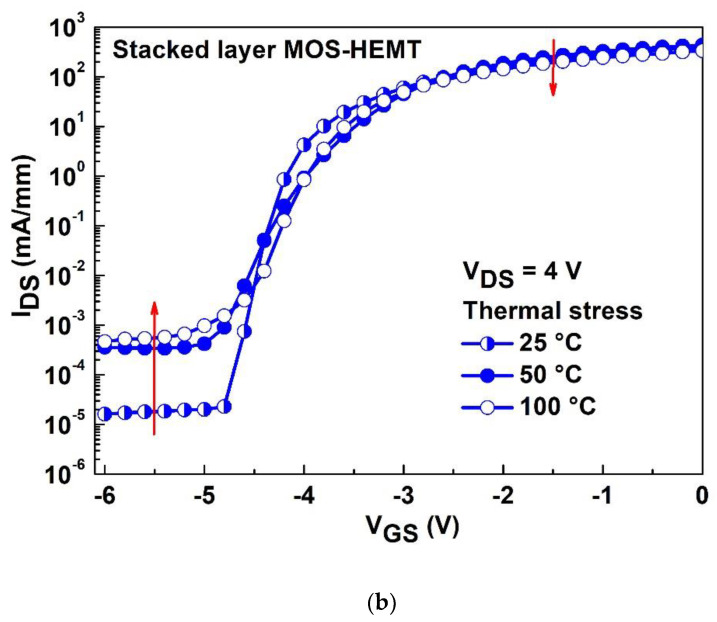
Subthreshold characteristics with various temperatures ranged from 25 °C to 100 °C for (**a**) the Schottky-gate HEMT and (**b**) the stacked layer MOS-HEMT.

Figure 7a,b show the temperature-dependent I_DS_-V_DS_ degradation characteristics with various temperatures ranged from room temperature (i.e., 25 °C) to 100 °C for the Schottky-gate HEMT and stacked layer MOS-HEMT, respectively. The temperature program ranged from 25 °C to 100 °C as the devices were measured with an Agilent B1500A semiconductor parameter analyzer. Degradation of the Schottky-gate HEMT and the stacked-layer MOS-HEMT is 47% and 26%, respectively. The R_on_ increased and the I_DS_ decreased at 0 V of V_GS_ for both devices as the temperature increased. Because of the good thermal conductivity of Al_2_O_3_, the heat dissipation is better and the degradation of the I_DS_ is smaller than those of the reference HEMT. Figure 8a,b show the subthreshold characteristics with various temperatures for the Schottky-gate HEMT and stacked-layer MOS-HEMT, respectively. The degraded on-state I_DS_ is due to the decreased saturation velocity or mobility [30] results from phonon scattering [31] that dominates the temperature effect at high drain current region, which is consistent with the result in Figure 7. On the contrary, the increased off-state I_DS_ with increasing temperature is owing to the ionized traps that dominate the temperature effect results in increasing the carrier concentration in the low drain current region. The threshold voltage shift to the positive bias direction with increasing temperature can be observed for both devices. Although the electrons can jump to the conduction band easily with increasing temperature, the Al_2_O_3_/ZrO_2_ stacked layer can block the tunneling current, leading to smaller variation in I_DS_ and threshold voltage shift compared with those of the reference HEMT in Figure 8a.

**Figure 9:**
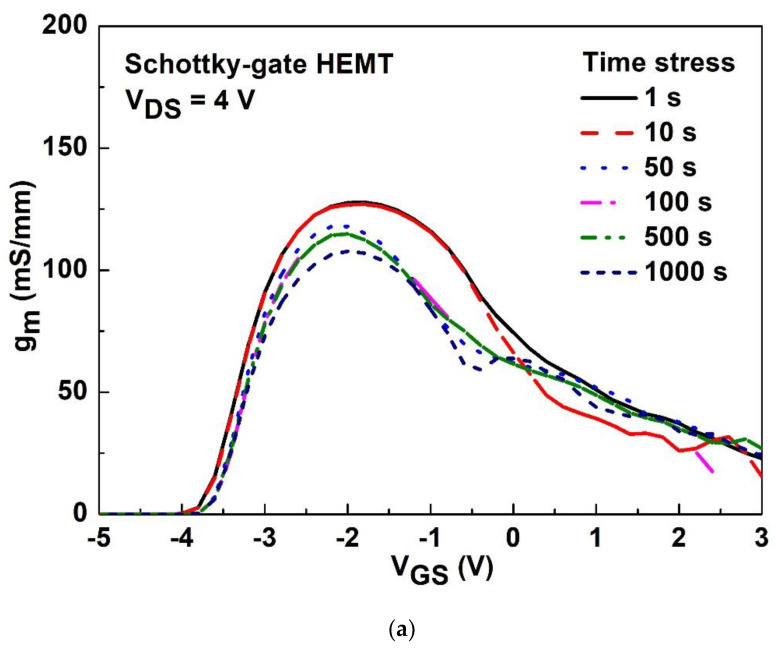

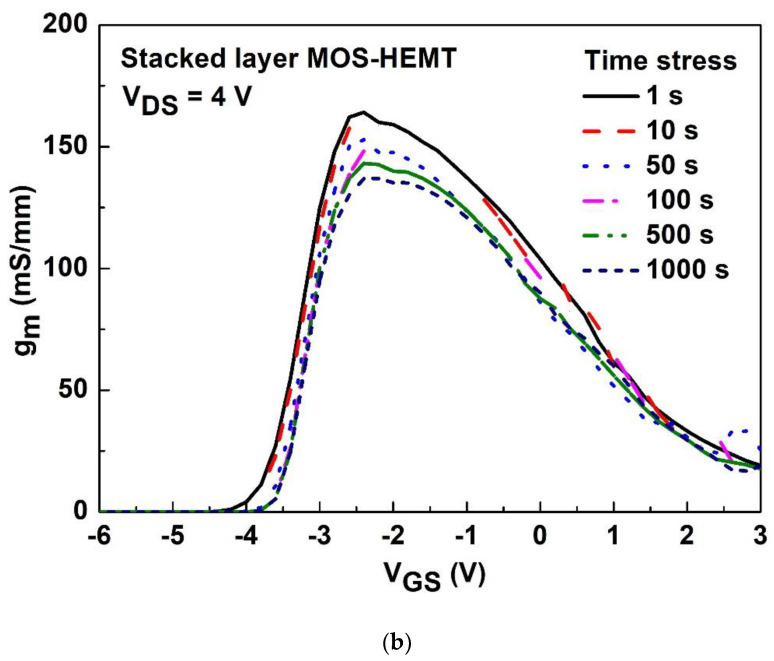
The g_m_ after stress at V_GS_ of 1 V and V_DS_ of 4 V with different stress time ranged from 1 s to 1000 s at room temperature for (**a**) the Schottky-gate HEMT and (**b**) the stacked layer MOS-HEMT.

**Figure 10:**
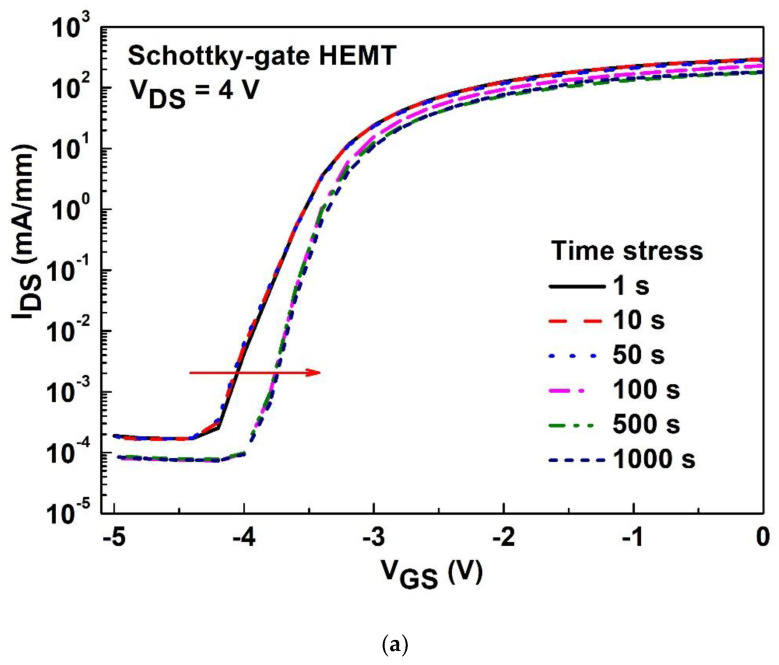

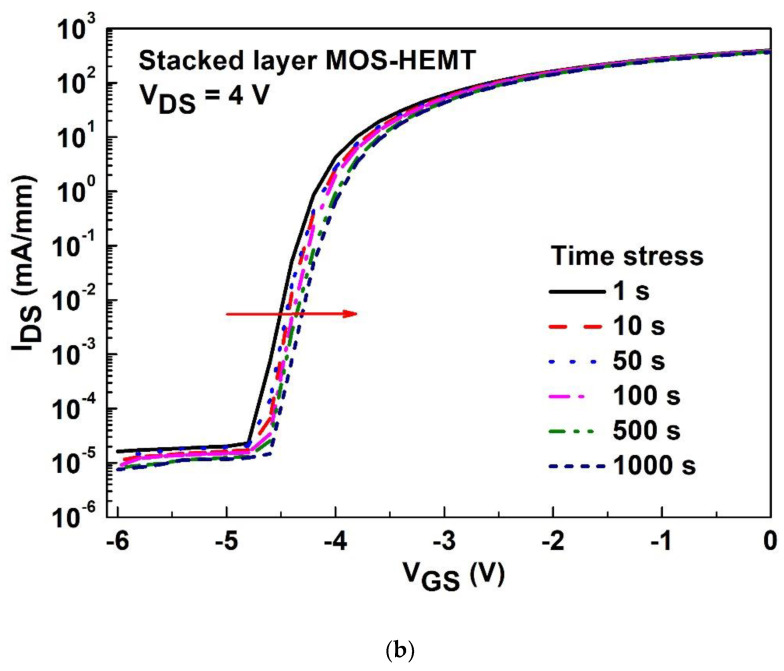
Subthreshold characteristics after stress at V_GS_ of 1 V and V_DS_ of 4 V with different stress time ranged from 1 s to 1000 s at room temperature for (**a**) the Schottky-gate HEMT and (**b**) the stacked-layer MOS-HEMT.

The time stress was biased at V_GS_ of 1 V and V_DS_ of 4 V, with times ranging from 1 s to 1000 s at room temperature. The monitoring of g_m_ after time stress was performed at V_DS_ of 4 V and V_GS_ sweeping from −6 to 3 V for the Schottky-gate HEMT and stacked-layer MOS-HEMT, respectively, as shown in Figure 9a,b. The degradation of peak g_m_ (16%) for both devices are almost the same; however, the g_m_ becomes somewhat unstable as the gate bias increases in Figure 9a. The subthreshold characteristics were measured after stress at V_GS_ of 1 V and V_DS_ of 4 V with different time ranged from 1 s to 1000 s at room temperature. The monitoring was conducted at V_DS_ of 4 V and V_GS_ sweeping from −6 to 0 V for the Schottky-gate HEMT and stacked-layer MOS-HEMT, respectively, as shown in Figure 10a,b. The aforementioned results in Figure 5 describing the defects or traps behavior implies that fewer carriers were easily trapped versus detrapped due to the high energy gap of Al_2_O_3_/ZrO_2_, which means the smaller degradation of I_DS_ or subthreshold current suggests that there are less interface states between the metal gate and GaN. In other words, the subthreshold current decreases with increasing time for both devices; however, a larger variation in threshold voltage shift is attributed to the capture/emission process of traps happening at the interface between the metal gate and GaN as shown in Figure 10a. Table 1 summarizes the dc characteristics of MOS-HEMTs with different stacked gate dielectrics in this work and by other researchers [5,12,21,23,25]. The dc characteristics presented in this work are comparable with those of other groups. The proposed Al_2_O_3_/ZrO_2_ stacked-gate dielectrics are reliable for device applications.

**Table 1:** A summary of the dc characteristics of MOS-HEMTs with different stacked gate dielectrics in this work and by other groups.

Group	This Work	[5]	[21]	[23]	[25]	[12]
**Gate oxide (nm)/** **Oxidation technique**	Al_2_O_3_/ZrO_2_ (1/12) RF co-sputter	SiN_x_/HfO_2_ (5/25) PEALD^+^ and RF sputter	Al_2_O_3_, Ga_2_O_3_/Gd_2_O_3_ (5/10) N_2_O plasma and electron-beam evaporation	Al_2_O_3_/ZrO_2_ (2/2) ALD^++^	Y_2_O_3_/HfO_2_ (1/12) PEALD^+^	Al_2_O_3_/ HfO_2_ (2/3) ALD^++^
**Mode**	D	E	E	D	D	D
**Gate length (µm)**	1	2	2	2	1	1
**Maximum I_DS_ (mA/mm)**	847	600	364	540	600	800
**R_on_ (Ω∙mm)**	3.82	~8.8	-	6.6	10.7	~5.0
**Peak g_m_** **(mS/mm)**	181@V_DS_ = 4 V	170@V_DS_ = 10 V	105@V_DS_ = 8 V	94	4.8@V_DS_ = 0.05 V	150@V_DS_ = 10 V
**Subthreshold swing (mV/dec)**	95	85	-	-	70	-
**I_ON_/I_OFF_**	3.3 × 10^7^	10^9^	-	10^9^	10^9^	-
**Dit (cm^−2^)**	2.44 × 10^11^	-	-	-	~10^12^	-

^+^ Plasma enhanced atomic layer deposition; ^++^ Atomic layer deposition.

## 4. Conclusions

This study demonstrated the improved electrical characteristics of AlGaN/GaN MOS-HEMT with Al_2_O_3_/ZrO_2_ stacked gate dielectrics. Improved electrical characteristics included suppressed gate leakage current, decreased flicker noise, increased high-frequency performance, better power performance, and enhanced stability after thermal stress or time stress. The gate leakage current can be reduced by three orders of magnitude due to the Al_2_O_3_/ZrO_2_ stacked layer, which also contributed to the lower flicker noise. To further understand the stability of the proposed device, thermal and time stresses were conducted. The thermally induced degradation of I_DS_ was smaller than that of reference HEMT. The variation of subthreshold characteristics caused by thermal or time stress was smaller than that of the reference case, showing the proposed Al_2_O_3_/ZrO_2_ stacked gate dielectrics are reliable for device applications.
